# Phenotype of apoptotic lymphocytes in children with Down syndrome

**DOI:** 10.1186/1742-4933-6-2

**Published:** 2009-03-06

**Authors:** Solaf M Elsayed, Ghada M Elsayed

**Affiliations:** 1 Genetics Unit, Pediatrics Department, Ain Shams University, Cairo, Egypt; 2 Clinical Pathology Department, National Cancer Institute, Cairo University, Egypt

## Abstract

**Background:**

Down syndrome (DS) is the most common and best-known chromosomal disorder and is associated with several other pathologic conditions including immunodeficiency which makes a significant contribution to morbidity and mortality. Various immunological theories and observations to explain the predisposition of individuals with DS to various infections have been published, one of which is increased apoptotic cells.

**Aim:**

The aim of this study was to identify the effect of apoptosis on both types of cells of specific immune response (T and B lymphocytes) in children with DS using Annexin V staining of phosphatidyserine (PS) as a specific marker of early apoptosis.

**Subjects and methods:**

The study included 17 children with karyotypically ascertained DS (7 males and 10 females). Their ages ranged from 4 months to 14 years with mean age of 5.7 ± 4.35 years. Seventeen age and sex matched healthy children were included in the study as controls. Patients or controls with infections were excluded from the study. Complete blood picture, immunophenotyping, analysis of apoptosis using Annexin V was done at National cancer Institute to all children included in this study.

**Results:**

Although CBC, differential count, relative and absolute number of CD^3+ ^and CD^16+ ^did not show significant differences between DS children and control group, the relative and the absolute size of apoptotic CD^3+ ^T lymphocytes, and the relative size of apoptotic CD^19+ ^B lymphocytes were significantly higher in DS children than in controls. On the other hand, no significant difference was detected as regards the absolute size of CD^19+ ^B lymphocytes in DS children and in controls

**Conclusion:**

our finding of increased early apoptotic cells (especially T cells) in DS children may emphasize the fact that the function of cells- and not their number- is main mechanism responsible for the impairment of the immune system in DS children and may further add to the known fact that cellular immunity is more severely affected than humoral immunity in these children. Further studies on apoptotic cellular phenotype in larger number of DS are needed

## Introduction

Down syndrome (DS) is the most common and best-known chromosomal disorder. It is associated with several pathologic conditions including immunodeficiency which makes a significant contribution to morbidity and mortality in these children. Various immunological theories and observations to explain the predisposition of individuals with DS to infections have been published [1-6]. One of the most interesting; is the "precocious aging" theory in which increased apoptosis is the main cornerstone [7]. Previous authors reported increased apoptotic cells in neurons and granulocytes of DS patients [8,9]. It has also been studied in the peripheral blood by means of electron microcopy, in situ nick translation (ISNT) and DNA electrophoresis [10]. These methods detects only late events of apoptosis (apoptotic body formation and fragmentation of DNA into oligonucleosomal fragments). Early apoptosis can be detected using Annexin V staining of phosphatidyserine (PS). This method has been used before by Corsi et al., 2003 who reported an increase in early apoptotic CD^3 ^lymphocytes in DS children compared to the controls [11]. However, the effect of apoptosis on both types of cells of specific immune response (T and B lymphocytes) was not studied before.

The aim of this study was to identify the effect of apoptosis on both types of cells of specific immune response (T and B lymphocytes) in children with DS using Annexin V staining of phosphatidyserine (PS) as a specific marker of early apoptosis.

## Subjects and methods

### Subjects

The study included 17 children with karyotypically ascertained DS (7 males and 10 females) from the genetics clinic, Ain Shams University. Their ages ranged from 4 months to 14 years with mean age of 5.7 years ± 4.3 years and a median of 7 years. Seventeen age and sex matched healthy children were included in the study as controls. Each patient had a karyotypically normal control from the same age and sex (controls were actually healthy patients' sibs to stabilize diet and other environmental factors that may affect immunity). Patients or controls with respiratory, urinary tract, gastrointestinal or other infections were excluded from the study. Complete blood picture and analysis of apoptosis were done in the National cancer Institute to all children included in this study.

### Methods

#### 1-Complete blood count and immunophenotyping

The relative and the absolute leukocyte counts were determined with a Sysmex SE-9500 hematology analyzer (Sysmex, Kobe, Japan). The region of lymphocyte population (lymphocyte gate), was set manually, based on the forward-scatter and side-scatter characteristics (Beckman Coulter flow cytometer, USA). The relative count of each lymphocyte subpopulation was expressed as a percentage within the total lymphocyte population. The absolute count of each lymphocyte subpopulation (CD^3+ ^and CD^19+ ^lymphocytes) was calculated from the relative count of the lymphocyte subpopulation, the relative count of the total lymphocyte population, and the absolute leukocyte count [12].

#### 2-Apoptosis in peripheral T- and B-lymphocytes

During apoptosis, externalization of phosphatidyserine (PS) and phosphatidylethanolamine is a hallmark of the changes in the cell surface. These phospholipids are normally sequestered within the cell surface on the cytoplasmic side of the plasma membrane. This occurs relatively early just after segmentation of the nucleus during which the cell membrane remains intact [13].

The permeability of the plasma membrane is a central difference between necrosis and apoptosis. Large molecular DNA binding dyes, such as propidium iodide (PI), can not enter intact cells because of their large size and without permebilization treatment, and do not label apoptotic cells until the final lysis stage. Annexin V, a Ca^2^-dependent phospholipids binding protein, which possesses high affinity for PS can be used specifically for detecting early apoptotic cells. When used with promidium iodide (PI), Annexin V staining allows the quantification of cells at early stages of apoptosis and the simultaneous identification of cell surface markers [14].

In this study, apoptosis in T- and B-lymphocytes was measured by staining with Fluorescein isothiocyanate (FITC) conjugated annexin V, and Propidium iodide (PI) using IQ products Phosphatidyl Serine Detection Kit (IQP-116F). Indotricarbocyanine (Cy5) coupled to Phycoerythrin (PE) conjugated anti-CD3 (PE-Cy5) IQP-519 and anti-CD19 (PE-Cy5) Dako-C7066 antibodies were used to identify apoptotic cell phenotype. Three-color flow cytometry analysis was performed on a BCKMAN Coulter equipped with a single 488 nm argon ion laser. At least 10,000 events were acquired for each sample. The voltages and compensation were set according to the standard procedure, using negative controls and tested cells stained in a single color or a combination of colors. The proportion of FITC+/PI-, corresponding to early apoptosis in T and B lymphocytes, was evaluated by gating for CD3-PE-Cy5 and CD19-PE-Cy5.

## Results

### 1-Complete blood picture (CBC) and differential count

Red blood cells, count, haemoglobin level, heamatocrite level, mean corpuscular volume, mean corpuscular heamoglobin, platelets and total leucocytic counts did not show significant difference between DS children and control group. No significant difference was also observed between the percentage and the absolute values of neutrophils, lymphocytes, monocytes or eosinophils between DS and normal children. The percentage of basophils was significantly higher in DS children but no significant difference was detected when absolute values were compared.

### 2-Lymphocyte population

Neither the absolute nor the relative values for CD^3+ ^T lymphocytes and CD^19+ ^B lymphocytes showed a significant difference between children with DS and controls. A slight lower figure was noted in absolute value of CD^19+ ^B lymphocytes in DS children (table 1).

**Table 1:** Lymphocyte population in DS children and controls

**Variable**		**DS** **(Mean ± SD)**	**Controls** **(Mean ± SD)**	**P-value**
**CD^3+ ^T lymphocytes**	**Percentage**	67.19 ± 11.81	62.11 ± 10.31	0.163
	
	**Absolute (×10^3^/μl)**	2.44 ± 3.05	2.16 ± 1.17	0.687

**CD^19+ ^B lymphocytes**	**Percentage**	13.20 ± 5.74	17.39 ± 7.27	0.136
	
	**Absolute (×10^3^/μl**)	0.40 ± 0.31	0.67 ± 0.61	0.076

### 3-Early apoptosis in peripheral blood T and B lymphocytes

**Figure 1:**
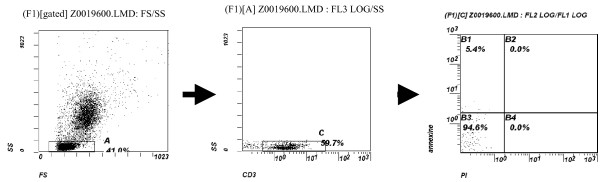
**Flow cytometric analysis of apoptotic CD^3+ ^T-lymphocytes in a healthy control**. From left to right: 1-dot plot showing gating on total lymphocytes, 2-dot plot showing gating on CD^3+ ^T-lymphocytes, 3-dot plot showing the proportion of early apoptotic cells in the upper left quadrant.

**Figure 2:**
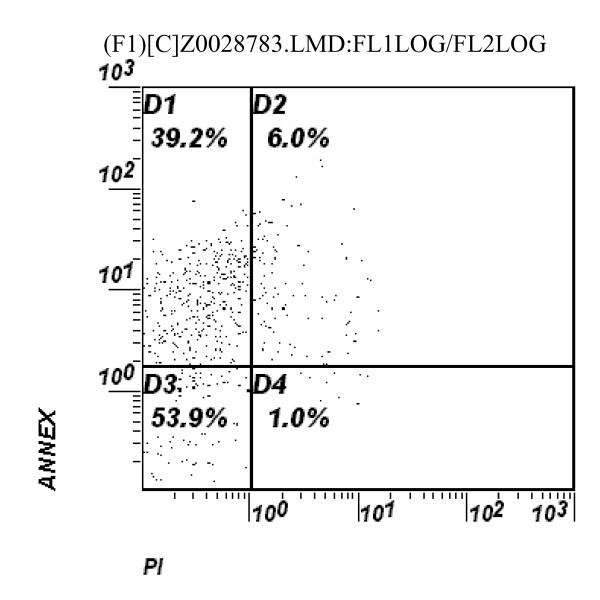
**Flow cytometric analysis of apoptotic CD^3+ ^positive T-lymphocytes in a child with Down syndrome**. The percentage of FITC+/PI-in the upper left quadrant (early apoptotic cells), and the FITC+/PI+ in the upper right quadrant (late apoptotic cells) as well as the FITC-/PI+ (necrotic) cells in the lower right quadrant after gating on CD^3+ ^cells in a DS child.

• CD^3+ ^T lymphocytes: Both relative and the absolute size of early apoptotic CD^3+ ^T lymphocytes, were significantly higher in DS children than in controls.

• CD^19+ ^B lymphocytes: The relative size of early apoptotic CD^19+ ^B lymphocytes were significantly higher in DS children than in controls, however, the absolute size was insignificantly higher in DS children compared to the controls, (table 2) and (figure 1, figure 2)

**Table 2:** Early apoptosis in peripheral blood T- and B – lymphocytes in DS children and controls.

	**Variable**	**DS** **(Mean ± SD)**	**Controls** **(Mean ± SD)**	**P-value**
**CD3**	**Percentage**	32.11 ± 13.57	9.65 ± 8.04	**0.001**
	
	**Absolute (×10>^3^/μl)**	0.79 ± 1.19	0.18 ± 0.13	**0.001**

**CD19**	**Percentage**	27.85 ± 16.68	13.54 ± 12.36	**0.022**
	
	**Absolute (×10^3^/μl**)	0.10 ± 0.07	0.08 ± 0.11	0.286

## Discussion

An impairment of both specific and non-specific immunity has been documented in patients with DS. Decreased neutrophil chemotaxis, leucocytes opsonization, and phagocytosis and leucocytes bactericidal activity were described in children with DS [15].

Several studies have focused their attention on the role of the thymus, and have described a variety of structural and anatomic alterations present in DS [16]. Studies of T-cell phenotype and function have frequently resulted in conflicting results, the overall evidence strongly pointed to a primary and profound impairment of T-cell mediated immunity [17].

In this work, neither the absolute nor the relative values for CD^3+ ^T lymphocytes and CD^19+ ^B lymphocytes showed a significant difference between children with DS and controls. Previous quantitative studies of peripheral blood T lymphocytes revealed a reduction, often quite small, in the percentage and/or absolute number of T lymphocytes, although normal proportions or numbers of T and B lymphocytes in DS children have also been reported [1,11].

Apoptosis, or programmed cell death, is a well-documented phenomenon in many cellular systems. It plays a key role in tissue and organ development during embryogenesis as well as in adult tissues during cell turn-over. It was suggested that apoptosis provides the mechanism for deletion of auto reactive T-cells in the thymus, low responsive B-cells in the germinal center, and of target cells attacked by cytotoxic T-lymphocytes and natural killer cells [13].

Previous studies evaluated apoptosis in peripheral blood of patients with DS by different methods but did not identify the phenotype of apoptotic cells [10]. In our work, both relative and absolute number of early apoptotic T cells (CD^3+ ^annexinV positive cells), were significantly lower in DS children while the absolute number of T-lymphocytes was insignificantly different. In other words, the function and not the number of T lymphocytes is the main mechanism responsible for the impairment of immunity in these children. This result supports the finding of Corsi et al., 2003 who explained this increase in apoptotic T cells by antigen overload combined with impairment of nutrient absorption (like zinc) secondary to altered function of the gastrointestinal mucosa [11]. Actually, decrease in number of apoptotic cells was observed after zinc supplementation in these children [10].

In our study, although both T and B cells showed increased apoptosis, B cells were less functionally impaired (as their absolute number was insignificantly different), which agrees with previous studies reporting that humoral immunity is less-strikingly impaired in these children [17] and necessitates further study on a larger scale of DS population to confirm this finding.

On the other hand, Roat et al., 2007 found that DS peripheral blood mononuclear cells (PBMNC) with altered mitochondrial function do not undergo apoptosis, and hypothesized that DS patients tend to maintain damaged cells, or that they have a higher capacity to repair functional damages. They added that it remains to be established if and how this phenomenon can be linked to the development of autoimmunity or neoplastic disorders. In contrast to their study, we studied only lymphocytes and not all PBMNC and that may explain the difference between the two studies [18].

Other authors implicate the absence of early expansion of T and B-lymphocytes in the peripheral blood of children with DS in the first year of life as the main cause of recurrent infections in early life of these children. They found also that T-lymphocyte subpopulation gradually approaches those of normal children over time which contradicts the theory of precocious aging. Nevertheless it does not explain the observed disturbance in the adaptive immune system in DS after the first year [12].

**In conclusion**, our finding of increased early apoptotic cells (especially T cells) in DS children may emphasize the fact that the function of cells- and not their number- is main mechanism responsible for the impairment of the immune system in DS children and may further add to the known fact that cellular immunity is more severely affected than humorral immunity in these children. Further studies on apoptotic cellular phenotype in larger number of DS are needed.

## Abbreviations

Cy5: Indotricarbocyanine; DS: Down syndrome; FITC: Fluorescein isothiocyanate; ISNT: in situ nick translation; PI: Propidium iodide; PBMNC: Peripheral blood mononuclear cells; PE: Phycoerythrin; PS: phosphatidyserine.

## Competing interests

The authors declare that they have no competing interests.

## Authors' contributions

SE: Designed the study, collected subjects' samples, analyzed and interpreted the data, and wrote the manuscript. GE: carried out CBC, immunophenotyping and apoptosis studies, and participated in analysis of data and writing the manuscript.

Both authors read and approved the final manuscript.
